# Retraction: Using mathematical models to evaluate germination rate and seedlings length of chickpea seed (*Cicer arietinum* L.) to osmotic stress at cardinal temperatures

**DOI:** 10.1371/journal.pone.0272196

**Published:** 2022-08-03

**Authors:** The PLOS ONE Editors

The *PLOS ONE* Editors retract this article [1] because it was identified as one of a series of submissions for which we have concerns about authorship, competing interests, and peer review. We regret that the issues were not addressed prior to the article’s publication.

All authors did not agree with the retraction.
